# Computerized Clinical Decision Support Systems for the Early Detection of Sepsis Among Pediatric, Neonatal, and Maternal Inpatients: Scoping Review

**DOI:** 10.2196/35061

**Published:** 2022-05-06

**Authors:** Khalia Ackermann, Jannah Baker, Marino Festa, Brendan McMullan, Johanna Westbrook, Ling Li

**Affiliations:** 1 Centre for Health Systems and Safety Research Australian Institute of Health Innovation Macquarie University Australia; 2 Kids Critical Care Research Department of Paediatric Intensive Care Children's Hospital at Westmead Sydney Australia; 3 Department of Immunology and Infectious Diseases Sydney Children's Hospital Randwick Sydney Australia; 4 Faculty of Medicine & Health University of New South Wales Sydney Australia

**Keywords:** sepsis, early detection of disease, computerized clinical decision support, patient safety, electronic health records, sepsis care pathway

## Abstract

**Background:**

Sepsis is a severe condition associated with extensive morbidity and mortality worldwide. Pediatric, neonatal, and maternal patients represent a considerable proportion of the sepsis burden. Identifying sepsis cases as early as possible is a key pillar of sepsis management and has prompted the development of sepsis identification rules and algorithms that are embedded in computerized clinical decision support (CCDS) systems.

**Objective:**

This scoping review aimed to systematically describe studies reporting on the use and evaluation of CCDS systems for the early detection of pediatric, neonatal, and maternal inpatients at risk of sepsis.

**Methods:**

MEDLINE, Embase, CINAHL, Cochrane, Latin American and Caribbean Health Sciences Literature (LILACS), Scopus, Web of Science, OpenGrey, ClinicalTrials.gov, and ProQuest Dissertations and Theses Global (PQDT) were searched by using a search strategy that incorporated terms for sepsis, clinical decision support, and early detection. Title, abstract, and full-text screening was performed by 2 independent reviewers, who consulted a third reviewer as needed. One reviewer performed data charting with a sample of data. This was checked by a second reviewer and via discussions with the review team, as necessary.

**Results:**

A total of 33 studies were included in this review—13 (39%) pediatric studies, 18 (55%) neonatal studies, and 2 (6%) maternal studies. All studies were published after 2011, and 27 (82%) were published from 2017 onward. The most common outcome investigated in pediatric studies was the accuracy of sepsis identification (9/13, 69%). Pediatric CCDS systems used different combinations of 18 diverse clinical criteria to detect sepsis across the 13 identified studies. In neonatal studies, 78% (14/18) of the studies investigated the Kaiser Permanente early-onset sepsis risk calculator. All studies investigated sepsis treatment and management outcomes, with 83% (15/18) reporting on antibiotics-related outcomes. Usability and cost-related outcomes were each reported in only 2 (6%) of the 31 pediatric or neonatal studies. Both studies on maternal populations were short abstracts.

**Conclusions:**

This review found limited research investigating CCDS systems to support the early detection of sepsis among pediatric, neonatal, and maternal patients, despite the high burden of sepsis in these vulnerable populations. We have highlighted the need for a consensus definition for pediatric and neonatal sepsis and the study of usability and cost-related outcomes as critical areas for future research.

**International Registered Report Identifier (IRRID):**

RR2-10.2196/24899

## Introduction

### Sepsis Identification

Sepsis, redefined in adults in 2016 as “life-threatening organ dysfunction caused by a dysregulated host response to infection” [1], was associated with an estimated 11 million deaths worldwide in 2017 [2]. Neonatal, pediatric, and obstetric populations are particularly vulnerable to developing sepsis [2-4].

Children aged <5 years accounted for approximately 40% of the estimated 50 million people diagnosed with sepsis in 2017 [2]. Furthermore, a recent report indicated that children aged <1 year have a considerably higher sepsis incidence rate compared with other age groups in Australia [5]. An estimated 28 neonatal sepsis cases occur per 1000 live births, with an associated mortality rate of 17.6% [4]. Survivors of pediatric sepsis have a substantial reduction in health-related quality of life compared with nonsepsis cases, with increased risk of hospital readmissions, cognitive impairment, and physical disability [6-9]. Similarly, surviving neonatal sepsis is associated with both short- and long-term neurodevelopmental delay and disability [10,11].

The most recent consensus definition of pediatric sepsis was presented in 2005, applicable to children from full-term birth to 18 years of age, and defined pediatric sepsis as modified “systemic inflammatory response syndrome (SIRS) in the presence of or as a result of suspected or proven infection” [12]. The definition of pediatric septic shock, a severe and often fatal progression of sepsis, was refined by the 2020 Surviving Sepsis Campaign guidelines to “severe infection leading to cardiovascular dysfunction (including hypotension, need for treatment with vasoactive medication, or impaired perfusion)” [13]. There is currently no formal definition of sepsis distinct to the neonatal population [14,15]; however, a recent systematic review of randomized controlled trials found neonatal sepsis to be most commonly defined by blood culture alone, followed closely by blood culture combined with clinical signs [16].

In the maternal population, a consensus definition for maternal sepsis was presented in 2017, defined as “organ dysfunction resulting from infection during pregnancy, child-birth, post-abortion, or post-partum period” [3]. The World Health Organization Global Maternal Sepsis Study [17] found the ratio of maternal infections in hospitalized women to be 70.4 (95% CI 67.7-73.1) women per 1000 live births. Furthermore, in 2014, a World Health Organization analysis indicated that 10.7% of maternal deaths between 2003 and 2009 were associated with sepsis [18]. Maternal sepsis also affects the health of the child and has been associated with serious complications, such as neonatal sepsis, spontaneous abortions, preterm births, and over 4.5 times the risk of death in the child [3,19,20].

Prompt initiation of treatment is critical for successful sepsis management [21-23]. The earlier sepsis is detected, the faster therapies can be initiated [24]. Therefore, early detection is key to improving patient outcomes. However, pediatric, neonatal, and maternal sepsis can be challenging to identify. Age-dependent physiological norms contribute to vague or nonspecific symptoms and extreme variation between patient presentations, making it difficult for clinicians to distinguish between benign conditions and more severe disease [3,15,25-28]. Recently, clinical tools, often as part of associated care bundles and clinical programs, have been developed to facilitate improved sepsis recognition, organ dysfunction assessment, and prediction of poor outcomes for pediatric (eg, pediatric sequential organ failure assessment [29], pediatric logistic organ dysfunction-2 score [30], and pediatric sepsis score [31]), neonatal (eg, neonatal sequential organ failure assessment [32]), and maternal sepsis (eg, modified obstetric early warning score [33] and sepsis in obstetrics score [34]). However, these tools typically rely on timely and regular vital sign monitoring by clinical staff to ensure that deteriorating patients are promptly detected [35,36].

### CCDS Systems

The widespread implementation of clinical information systems has allowed for sepsis recognition tools to be integrated into computerized clinician decision support (CCDS) systems [37,38] to assist clinical staff with decision-making [39]. In particular, CCDS systems can be used to improve the early detection of sepsis by monitoring patient data and automatically alerting when a patient shows signs consistent with sepsis [36]. Over the last 20 years, 2 types of CCDS systems have been developed: knowledge-based CCDS using preprogrammed rules [39] and adaptive systems using machine learning and artificial intelligence techniques [40]. This review is focused only on knowledge-based CCDS systems.

### Research Questions and Aims

Despite the critical importance of sepsis detection, there is a paucity of research on pediatric, neonatal, and maternal sepsis recognition tools [14,15,17,37]. In this scoping review, we mapped the available research investigating the use of knowledge-based CCDS systems for the early detection of sepsis in pediatric, neonatal, and maternal inpatients to provide an overview of the field and identify knowledge gaps for future research. Specifically, we aimed to (1) scope the study contexts, designs, and research methods used; (2) summarize the study outcomes investigated; and (3) map the range of CCDS system designs and implementation features, such as the clinical criteria for sepsis.

## Methods

### Overview

A protocol detailing the methodology of this scoping review has been previously published [41]. This review follows the PRISMA-ScR (Preferred Reporting Items for Systematic Reviews and Meta-Analyses Extension for Scoping Reviews) statement [42]. A completed PRISMA-ScR checklist can be found in Multimedia Appendix 1.

### Study Selection

To identify relevant studies, we used a broad 3-step strategy [41], during which an experienced librarian was consulted. The final search strategy combined terms for sepsis, clinical decision support, and early detection, excluding terms for artificial intelligence, and was used to search MEDLINE, Embase, CINAHL, Cochrane, Latin American and Caribbean Health Sciences Literature (LILACS), Scopus, Web of Science, OpenGrey, ClinicalTrials.gov, and ProQuest Dissertations and Theses Global (PQDT). The search strategy used for MEDLINE is presented in Multimedia Appendix 2. The search was conducted in September 2020.

**Figure 1:**
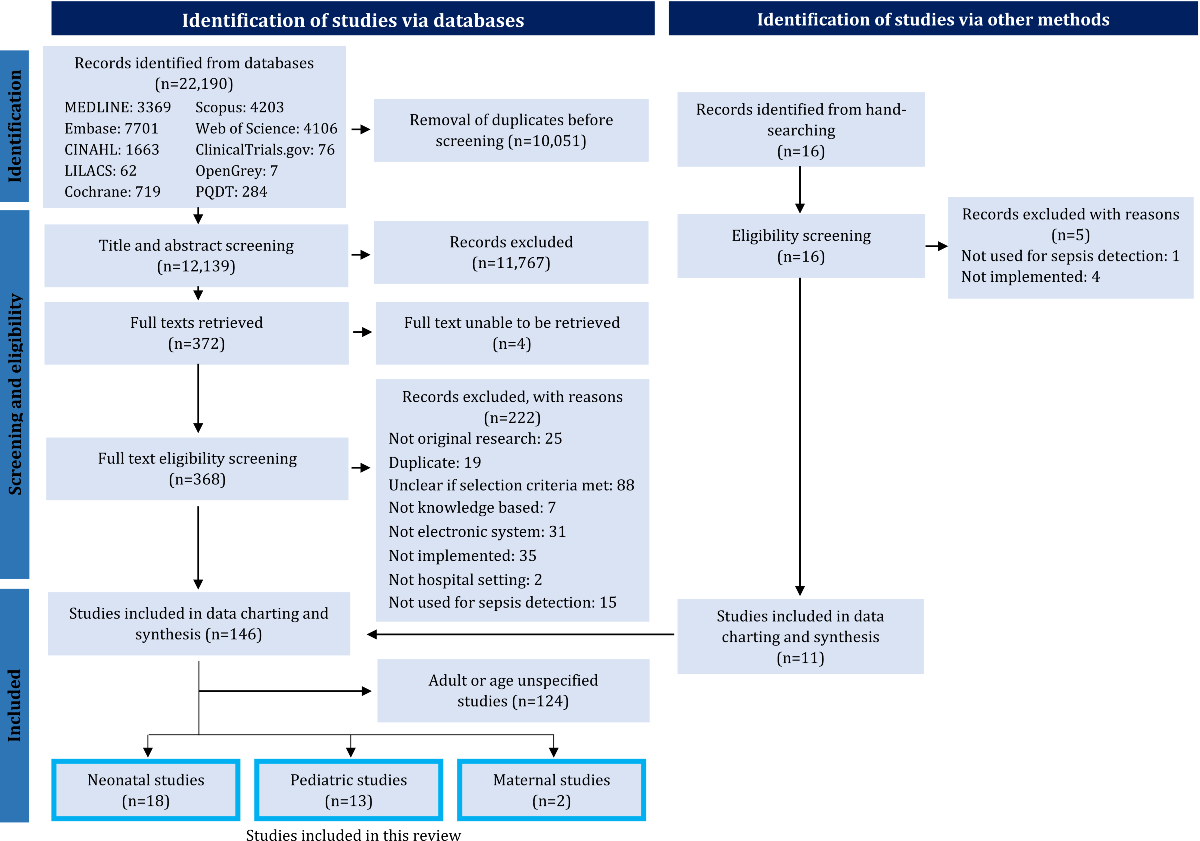
Flowchart of the search results and screening process. LILACS: Latin American and Caribbean Health Sciences Literature; PQDT: ProQuest Dissertations and Theses.

The search results were exported to an EndNote X9 (Clarivate) library. After deduplication, 2 reviewers (KA and JB) independently performed title, abstract, and full-text screening using the eligibility criteria reported in our protocol [41]. The reference lists of relevant systematic reviews and salient papers were manually searched by one reviewer (KA) with a second reviewer (JB) double-checking their inclusion to identify any further studies. Any disagreements were resolved through discussion or consultation with a third reviewer (LL). A PRISMA (Preferred Reporting Items for Systematic Reviews and Meta-Analyses) diagram visually representing this process is presented in Figure 1.

A total of 2 reviewers (KA and JB) independently piloted title and abstract screening with a random selection of 25 articles and full-text screening with a random selection of 10 articles. The results were discussed with a third reviewer (LL) to ensure consensus before undertaking the full screen. The 2 reviewers (KA and JB) had 100% agreement in the title and abstract pilot screen, 97.6% agreement in title and abstract screening, 60% agreement in the pilot full-text screen, and 77.4% agreement in full-text screening. Both peer-reviewed journal articles and gray literature studies, such as conference abstracts and theses, were included in this review. The gray literature that was later published as a peer-reviewed article was removed. Studies reporting the same methods and study cohorts but measuring different outcomes were included.

We chose to publish the results of this review in 2 manuscripts separated by patients’ age, given the distinct sepsis presentations and pathophysiology of pediatric, neonatal, and maternal patients compared with adults [3,28,43]. The results of the review investigating adult CCDS systems have been published previously [44].

### Data Charting

The form used for data charting was designed using Microsoft Access based on the data charting form previously used for adult studies [44]. The original version was refined based on sample data extracted from 2 pediatric, 2 neonatal, and 1 maternal study. The remaining studies were charted by a single reviewer (KA), with a sample of studies checked by a second reviewer (JB), and ongoing consultation with a third reviewer (LL). We accepted any definition of the charted items, as detailed in the studies.

The final form abstracted data based on all 3 aims and included all components, as listed in our protocol [41], with some minor adjustments, as presented in Multimedia Appendix 3 [45-51]. The outcomes listed comprised (1) outcomes reported in the aims, methods, and results and (2) outcomes from the study sections that met our inclusion criteria [41]. The following were excluded: (1) outcomes mentioned in the methods or introduction but not in the results; (2) analysis of demographic or clinical features not specifically identifying the performance of the alert, unless they were the only outcome or the main outcome reported; (3) outcomes not discussed in the aims or methods and not included in the main results tables; and (4) balancing and process outcome measures. We distinguished *live* CCDS as systems that were implemented and actively alerting and *silent* CCDS as systems that were implemented and running with alerts muted.

### Analyzing and Reporting the Results

The abstracted data were analyzed through a narrative review, with accompanying statistical summaries organized by population group and aims. Tables were created using frequency counts and percentages to summarize the data and produce graphical figures where appropriate. The results are presented separately for the journal articles and conference abstracts.

The charted data demonstrated substantial diversity; hence, individual categories were grouped to allow for meaningful analysis. We have included a breakdown of what is included in each group in Multimedia Appendix 4.

### Ethics Approval

This scoping review used data collected from published studies (including publicly available gray literature). No individual patient was involved, and only aggregate-level data were presented; hence, ethical approval or consent to participate was not required.

## Results

### Study Characteristics

A database search returned 22,190 results. After deduplication, 12,139 studies were included for title and abstract screening. The full texts of 368 articles were screened, and 146 studies were identified for inclusion in the review. Manual searching identified a further 11 records. Of the 157 included studies, 33 (21%) [52-84] investigated pediatric, neonatal, and maternal populations. In comparison, 124 (79%) studies examined adult or unspecified age (assumed adult) inpatient populations (Figure 1). Thus, pediatric, neonatal, and maternal studies only represented 8.3% (13/157), 11.5% (18/157), and 1.3% (2/157) of the total studies, respectively. This process is visually presented in a PRISMA flowchart, as shown in Figure 1. A table detailing the main characteristics of the 33 included studies is presented in Multimedia Appendix 5 [52-84].

### Pediatric Studies

**Figure 2:**
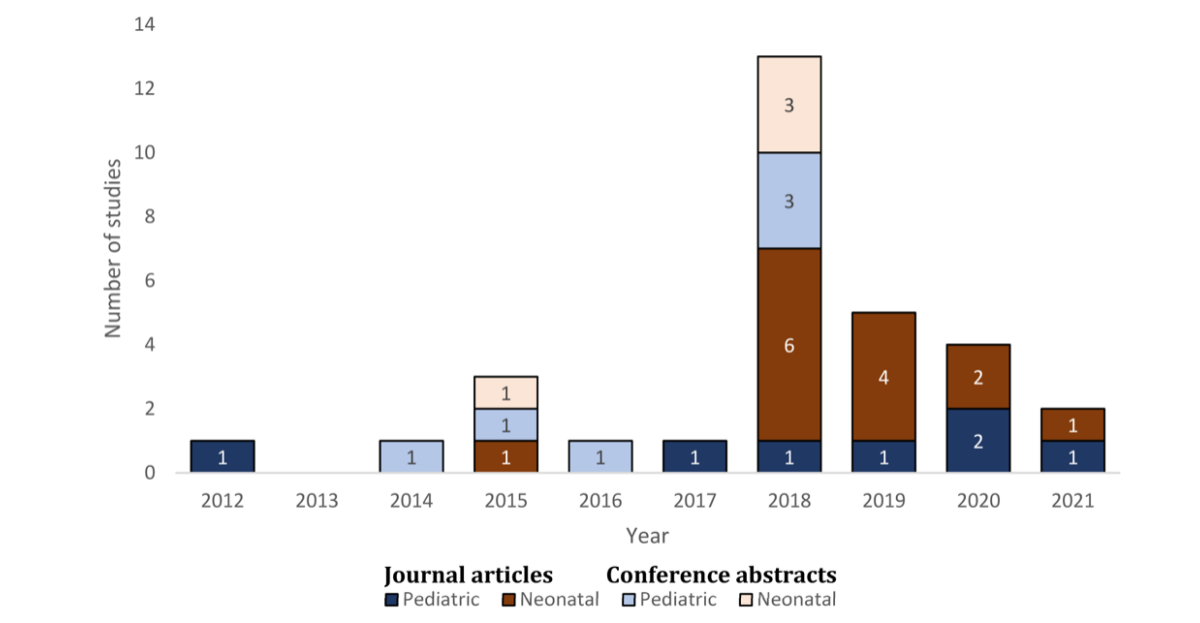
Studies investigating neonatal and pediatric computerized clinician decision support systems by year, population, and publication type.

Of the 13 studies investigating pediatric CCDS systems, 7 (54%) were journal articles and 6 (46%) were conference abstracts (Table 1). All studies were published in 2012 or later, with most journal articles (6/7, 86%) published after 2016 (Figure 2). Of the 13 studies, 11 (85%) were conducted in the United States, whereas the remaining 2 (15%) studies did not specify in which country they were conducted [64,73] (Multimedia Appendix 6). Of the 13 studies, 12 (92%) were conducted in children’s hospitals, whereas the remaining study [58] was conducted at a general hospital. All studies used quantitative methods, with the principal study design split between single cohort and before-after studies (Table 1).

**Figure 3:**
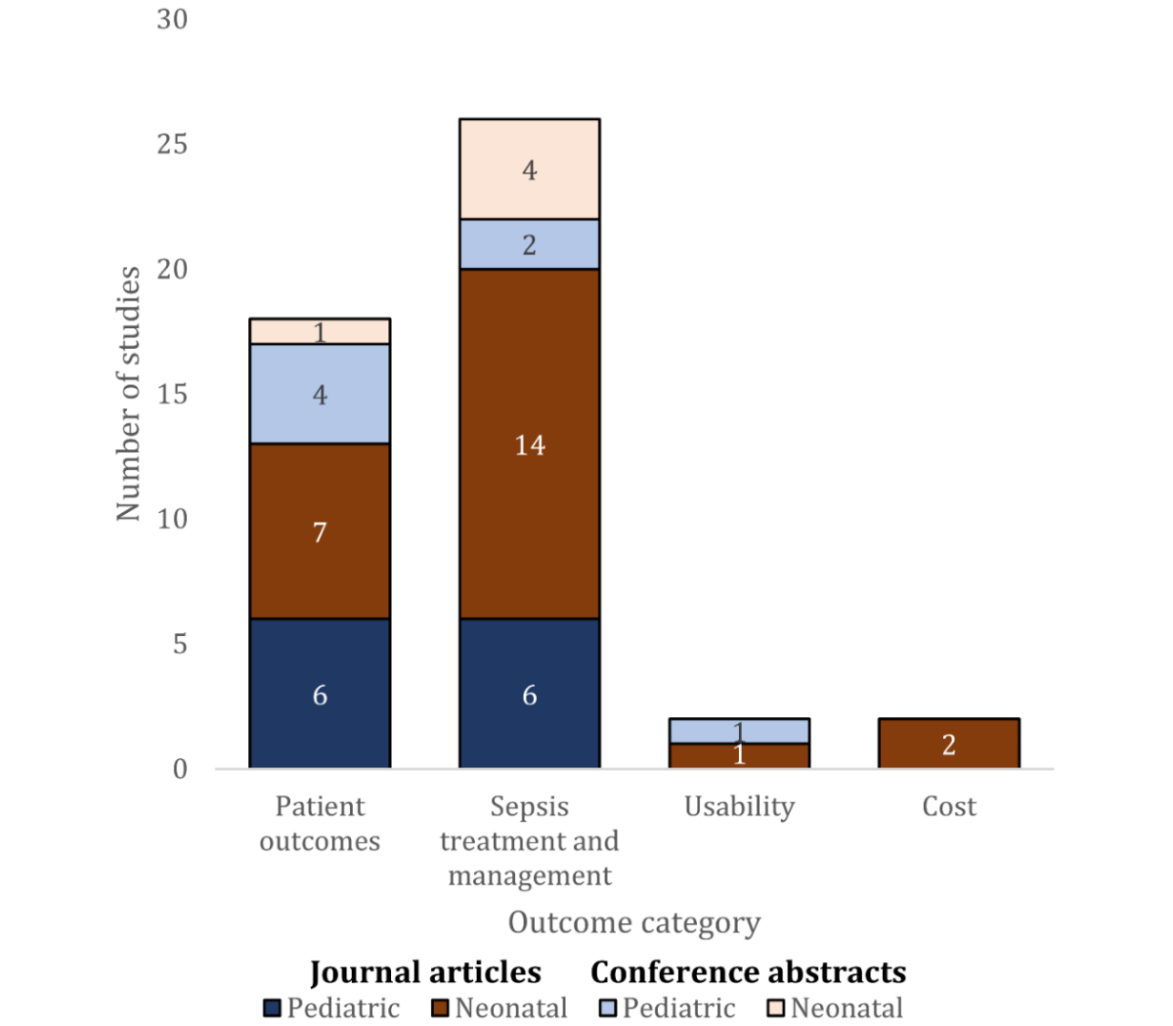
Outcome categories reported by studies by publication type and population.

The most common outcomes investigated were patient outcomes and sepsis treatment and management outcomes (Figure 3). Only 1 (8%) conference abstract [58] investigated an outcome related to the CCDS system usability, and none of the studies investigated pediatric CCDS-related cost outcomes (Figure 3). The most commonly investigated patient outcome was sepsis identification (9/13, 69%; Table 1). Pediatric CCDS systems were compared with the gold standard to measure the extent to which they identified sepsis. The gold standard definition used to determine true sepsis cases differed between studies, with 13 different definitions used to define sepsis across 9 studies (Table 1). Similarly, the method used to identify gold standard cases varied across studies: 38% (5/13) performed a chart review, 8% (1/13) prospectively screened patients, 8% (1/13) applied a manual screening tool, 8% (1/13) performed both a chart review and screened patients, and 8% (1/13) did not specify.

The main characteristics of the investigated pediatric CCDS systems are presented in Table 2. Most commonly, pediatric CCDS systems were live (10/13, 77%), homegrown (11/13, 85%), alerted via the electronic health record (6/13, 46%), and responded to by nurses (6/13, 46%) and other clinicians (5/13, 38%; Table 2).

The criteria used by the CCDS systems to identify sepsis cases are summarized in Table 3. In general, a diverse range of criteria was used to identify suspected sepsis cases, with 18 clinical criteria used across 9 pediatric CCDS systems in 8 studies included in this review. The remaining 5 pediatric studies [73,74,80,82,83], all conference abstracts, did not specify the CCDS system criteria used for sepsis case identification and were not included in Table 3. A total of 2 particular systems appear to be the subject of more than one study: the first in the studies by Dewan et al [61] and Vidrine et al [81] and the second in the studies by Stinson et al [77] and Viteri et al [82]. One journal article [64] is counted twice in Table 3, as it contains 2 separate electronic CCDS systems with different criteria: one with automated continuous screening and the other with clinician-initiated screening.

**Table 1:** Context and outcome characteristics for pediatric studies.

Study characteristics					Number of studies by publication				Total^a^
					Journal articles		Conference abstracts		
Subtotal, n					7		6		13
**Principal study type, n (%)**									
		Single cohort			3 (43)	4 (67)		7 (54)	
		Before-after			4 (57)	2 (33)		6 (46)	
**Setting, n (%)**									
		Hospital wide^b^			0 (0)	2 (33)		2 (15)	
		Emergency department			4 (57)	1 (17)		5 (38)	
		Intensive care unit			2 (29)	0 (0)		2 (15)	
		Inpatient units			1 (14)	3 (50)		4 (31)	
**Number of participants, n (%)**									
		≤100			1 (14)	2 (33)		3 (23)	
		101-10,000			1 (14)	2 (33)		3 (23)	
		10,001-100,000			2 (29)	1 (17)		3 (23)	
		>100,000			2 (29)	0 (0)		2 (15)	
		Unspecified			1 (14)	1 (17)		2 (15)	
**Funding, n (%)**									
		Yes (noncommercial)			2 (29)	0 (0)		2 (15)	
		No			2 (29)	0 (0)		2 (15)	
		Unspecified			3 (43)	6 (100)		9 (69)	
**Outcomes, n (%)**									
	**Patient outcomes**								
		**Sepsis identification**			5 (71)	4 (67)		9 (69)	
			**Gold standard definition^c^**						
				Goldstein et al [12]	2 (29)	0 (0)		2 (15)	
				American Academy of Pediatrics Sepsis Collaborative tool [85]	1 (14)	0 (0)		1 (8)	
				Clinician discretion	3 (43)	2 (33)		5 (38)	
				Improving Pediatric Sepsis Outcomes definition [86]	1 (14)	0 (0)		1 (8)	
				International Classification of Diseases codes	1 (14)	0 (0)		1 (8)	
				Not specified	1 (14)	2 (33)		3 (23)	
		Other			4 (57)	1 (17)		5 (38)	
	**Sepsis treatment or management, n (%)**								
		Timeliness of alert or intervention			3 (43)	1 (17)		4 (31)	
		Other			6 (86)	1 (17)		7 (54)	
	**Usability, n (%)**								
		Satisfaction			0 (0)	1 (17)		1 (8)	

^a^The percentages were calculated from the number of pediatric studies (n=13). As some studies reported multiple outcomes for each category, there were more than 13 outcomes in some categories, and therefore, the percentages add to more than 100%.

^b^If the study setting was not explicitly stated, it was assumed to be hospital wide.

^c^Some studies have used multiple definitions of sepsis as part of their gold standard.

**Table 2:** Computerized clinical decision support characteristics in pediatric studies.

CCDS^a^ characteristics				Number of studies by publication				Total^b^
				Journal articles		Conference abstracts		
Subtotal, n				7		6	13	
**CCDS type, n (%)**								
	Homegrown^c^			6 (86)		5 (83)	11 (85)	
	**Commercial, n (%)**			0 (0)		1 (17)	1 (8)	
		Epic monitor	0 (0)		1 (17)		1 (8)	
	Unspecified			1 (14)		0 (0)	1 (8)	
**Silent or live^d^, n (%)**								
	Live			5 (71)		5 (83)	10 (77)	
	Silent			1 (14)		1 (17)	2 (15)	
	Both (pre or post)			1 (14)		0 (0)	1 (8)	
**Related interventions, n (%)**								
	None			2 (29)		5 (83)	7 (54)	
	Response team			4 (57)		1 (17)	5 (38)	
	Education and information resources			3 (43)		1 (17)	4 (31)	
	Order sets			3 (43)		1 (17)	4 (31)	
	Sepsis protocol			1 (14)		1 (17)	2 (15)	
	Other			2 (29)		1 (17)	3 (23)	
**Responding personnel, n (%)**								
	Nurses			6 (86)		0 (0)	6 (46)	
	Other clinicians			3 (43)		2 (33)	5 (38)	
	Response team			0 (0)		2 (33)	2 (15)	
	Not specified			0 (0)		3 (50)	3 (23)	
**Alert delivery, n (%)**								
	Electronic health record			6 (86)		0 (0)	6 (46)	
	Emergency department tracking board			1 (14)		0 (0)	1 (8)	
	Not specified			0 (0)		6 (100)	6 (46)	

^a^CCDS: computerized clinical decision support.

^b^The percentages were calculated from the number of pediatric studies (n=13). As some studies reported multiple characteristics for each category, there were more than 13 characteristics in some categories; therefore, the percentages add to more than 100%.

^c^Homegrown CCDS systems are defined as CCDS systems that have been designed by the institution implementing them, rather than commercially available systems [41].

^d^A *live* CCDS system is a system that is implemented and being used by clinicians in real time during the study. Silent systems are systems that have been implemented but do not alert clinicians during the study and thus do not influence treatment.

**Table 3:** Clinical criteria used by pediatric computerized clinical decision support (CCDS) systems for sepsis identification.

	Study									
	Balamuth et al, 2017 [55]	Cruz et al, 2012 [59]	Dewan et al, 2020 [61]	Eisenberg et al, 2021 [64] (clinician-initiated)	Eisenberg et al, 2021 [64] (automated)	Lloyd et al, 2018 [71]	Stinson et al, 2019 [77]	Vidrine et al, 2020 [81]	Coffman et al, 2018^a^ [58]	Total, n (%^b^)
Temperature	✓	✓	✓	✓	✓	✓	✓	✓	✓	9 (69)
Capillary refill or perfusion	✓	✓	✓	✓		✓	✓	✓		7 (54)
Mental status	✓	✓	✓	✓		✓	✓	✓		7 (54)
Heart rate	✓	✓		✓	✓	✓	✓			6 (46)
Hypotension	✓		✓	✓		✓	✓	✓		6 (46)
High-risk patient	✓	✓		✓		✓	✓			5 (38)
Pulse assessment			✓	✓		✓	✓	✓		5 (38)
Skin assessment			✓	✓		✓	✓	✓		5 (38)
Respiratory rate				✓	✓	✓	✓			4 (31)
Infection concern, change in clinical or sepsis risk	✓			✓		✓				3 (23)
Blood culture order			✓					✓		2 (15)
Leukocyte count					✓					1 (8)
Cardiac organ dysfunction					✓					1 (8)
Noncardiac organ dysfunction					✓					1 (8)
Change in Pediatric Early Warning Score									✓	1 (8)
Family concern									✓	1 (8)
Vital sign change									✓	1 (8)
Patient risk change									✓	1 (8)

^a^This study is a conference abstract, and the other 8 studies are journal articles.

^b^The percentages were calculated from the number of pediatric studies (n=13).

### Neonatal Studies

Of the 18 articles investigating neonatal CCDS systems, 14 (78%) were journal articles and 4 (22%) were conference abstracts. All studies were published in 2015 or later, with most published in 2018 (n=9; Figure 2). Overall, 61% (11/18) of the studies were conducted in the United States, 11% (2/18) were conducted in the Netherlands, 11% (2/18) did not specify location, and 1 (6%) study each was set in Australia, Israel, and the United Kingdom (Multimedia Appendix 6). All neonatal studies used quantitative methods to investigate the CCDS systems. A total of 89% (16/18) of studies were single site, with the remaining 11% (2/18) of studies involving 4 [66] and 2 sites [69]. The gestational age range of neonates included in these studies was quite diverse, with 35 weeks and older being the most common inclusion threshold (Table 4).

The most common outcome used to investigate neonatal CCDS systems was sepsis treatment and management outcomes, followed by patient outcomes (Figure 3; Table 4). CCDS-related usability and cost outcomes were only investigated by 1 and 2 studies, respectively [53,56,66] (Figure 3; Table 4). Of the sepsis treatment and management outcomes, antibiotics-related outcomes were reported most frequently (15/18, 83%; Table 4). Table 5 reports the main characteristics of the neonatal CCDS systems. Notably, most studies investigated early-onset sepsis (15/18, 83%) using the neonatal early-onset sepsis risk calculator developed by the Kaiser Permanente team [87-89] (14/18, 78%).

**Table 4:** Context and outcome characteristics in neonatal studies.

Study characteristics				Number of studies by publication				Total^a^
				Journal articles		Conference abstracts		
Subtotal, n				14		4		18
**Principal study type, n (%)**								
	Single cohort			3 (21)		3 (75)		6 (33)
	Before-after			9 (64)		1 (25)		10 (56)
	Interrupted time series			2 (14)		0 (0)		2 (11)
**Setting, n (%)**								
	Hospital wide^b^			4 (29)		2 (50)		6 (33)
	Nursery			7 (50)		2 (50)		9 (50)
	ICU^c^			3 (21)		0 (0)		3 (17)
**Number of participants, n (%)**								
	≤100			0 (0)		1 (25)		1 (6)
	101-1000			5 (36)		1 (25)		6 (33)
	1001-10,000			6 (43)		0 (0)		6 (33)
	>10,001			2 (14)		0 (0)		2 (11)
	Unspecified			1 (7)		2 (50)		3 (17)
**Age of included neonates, n (%)**								
	<33 weeks gestation			1 (7)		0 (0)		1 (6)
	≥34 weeks gestation			3 (21)		1 (25)		4 (22)
	≥35 weeks gestation			4 (29)		1 (25)		5 (28)
	≥36 weeks gestation			2 (14)		0 (0)		2 (11)
	>37 weeks gestation			1 (7)		0 (0)		1 (6)
	First month of life			1 (7)		0 (0)		1 (6)
	Unspecified			2 (14)		2 (50)		4 (22)
**Funding, n (%)**								
	Yes (noncommercial)			1 (7)		0 (0)		1 (6)
	No			7 (50)		0 (0)		7 (39)
	Unspecified			6 (43)		4 (100)		10 (56)
**Outcomes, n (%)**								
	**Patient outcomes**							
		ICU admission	4 (29)		0 (0)		4 (22)	
		Length of stay	3 (21)		1 (25)		4 (22)	
		Other	4 (29)		1 (25)		5 (28)	
	**Sepsis treatment or management**							
		Antibiotics	12 (86)		3 (75)		15 (83)	
		Laboratory evaluation	8 (57)		3 (75)		11 (61)	
		Timeliness of alert or intervention	2 (14)		0 (0)		2 (11)	
		Sepsis guideline compliance	2 (14)		0 (0)		2 (11)	
		Other	4 (29)		1 (25)		5 (28)	
	**Usability**							
		Effectiveness	1 (7)		0 (0)		1 (6)	
	Cost			2 (14)		0 (0)	2 (11)	

^a^The percentages were calculated from the number of neonatal studies (n=18). As some studies have reported multiple outcomes for each category, there were more than 18 outcomes in some categories; therefore, the percentages add to more than 100%.

^b^If the study setting was not explicitly stated, it was assumed to be hospital wide.

^c^ICU: intensive care unit.

**Table 5:** Computerized clinical decision support characteristics in neonatal studies.

CCDS^a^ characteristics			Number of studies by publication				Total^b^
			Journal articles		Conference abstracts		
Subtotal, n			14		4		18
**Type of sepsis, n (%)**							
	Early-onset sepsis	12 (86)		3 (75)		15 (83)	
	Late-onset sepsis	1 (7)		0 (0)		1 (6)	
	Sepsis	1 (7)		1 (25)		2 (11)	
**General CCDS criteria, n (%)**							
	Kaiser Permanente early-onset sepsis risk [89]	12 (86)		2 (50)		14 (78)	
	Epic Monitor [65]	1 (7)		1 (25)		2 (11)	
	RALIS [69]	1 (7)		0 (0)		1 (6)	
	Not specified	0 (0)		1 (25)		1 (6)	
**Silent or live^c^, n (%)**							
	Live	12 (86)		4 (100)		16 (89)	
	Silent	1 (7)		0 (0)		1 (6)	
	Both (pre or post)	1 (7)		0 (0)		1 (6)	
**Related interventions, n (%)**							
	Education and information resources	8 (57)		1 (25)		9 (50)	
	None	4 (29)		3 (75)		7 (39)	
	Sepsis protocol	4 (29)		0 (0)		4 (22)	
	Order sets	2 (14)		0 (0)		2 (11)	
	Other	5 (36)		1 (25)		6 (33)	
**Responding personnel, n (%)**							
	Nurses	4 (29)		1 (25)		5 (28)	
	Other clinicians	10 (71)		0 (0)		10 (56)	
	Paramedics	1 (7)		1 (25)		2 (11)	
	Not specified	2 (14)		2 (50)		4 (22)	
**Alert delivery, n (%)**							
	Calculated by personnel	10 (71)		3 (75)		13 (72)	
	Other	2 (14)		0 (0)		2 (11)	
	Not specified	2 (14)		1 (25)		3 (17)	

^a^CCDS: computerized clinical decision support.

^b^The percentages were calculated from the number of neonatal studies (n=18). As some studies have reported multiple characteristics for each category, there were more than 18 characteristics, therefore, the percentages add to more than 100%.

^c^A *live* CCDS system is a system that is implemented and being used by clinicians in real time during the study. Silent systems are systems that have been implemented but do not alert clinicians during the study and thus do not influence treatment.

### Maternal Studies

Only 2 studies—those by Davis et al [60] and Blumenthal et al [57]—have investigated CCDS systems for sepsis in pregnant or immediately postpartum populations. Both studies were abstracts and used quantitative methods. Blumenthal et al [57] used a before-after study design, whereas Davis et al [60] did not provide sufficient information for the study design to be determined. Davis et al [60] conducted a single-site, hospital-wide study in the United States, and Blumenthal et al [57] conducted a study at 3 sites but did not specify in which country. None of the studies reported on the number of participants. To identify maternal sepsis, Davis et al [60] used the obstetric-adjusted systemic inflammatory response syndrome (SIRS) criteria (comprising SIRS with the addition of fetal heart rate) plus organ dysfunction, whereas Blumenthal et al [57] used a maternal early warning score (comprising temperature plus heart rate, altered mental state, respiratory rate, and mean arterial pressure). Both studies investigated sepsis treatment and management outcomes, with Blumenthal et al [57] additionally investigating patient outcomes.

## Discussion

### Principal Findings

This review comprehensively scoped the current literature on CCDS systems for early detection of sepsis in pediatric, neonatal, and maternal hospital populations. Overall, our findings highlight the scarcity of studies in these unique populations when compared with the general adult population, representing only 21% (33/157) of studies. Furthermore, only 64% (21/33) of studies were peer-reviewed journal articles. Given the high burden of sepsis in pediatric, neonatal, and maternal patients, this comparatively small number of studies is concerning [2-4,18] and underlines the critical need for future high-quality research into CCDS systems for these vulnerable populations. However, the rapid expansion of this field in recent years is encouraging, with all 33 studies published in the last 10 years and the majority (26/33, 79%) published in the last 5 years.

### Pediatric Sepsis

Our findings emphasize the variability in pediatric studies that have evaluated the use of sepsis CCDS systems. In particular, we found great variability across the clinical criteria used for pediatric sepsis identification, with 18 different clinical criteria used in numerous combinations across 8 studies (Table 3). Furthermore, a range of gold standard definitions was applied, of which the most common was clinician discretion rather than published tools [12,85,86], highlighting the lack of a consensus definition and tool for pediatric sepsis identification. Hospital settings varied widely between studies, and numerous related interventions were implemented alongside the pediatric CCDS, with few similarities. This variability makes it difficult to compare studies and draw generalized conclusions from the literature. All studies were single cohort or before-after studies, highlighting the need for more robust study designs to provide stronger evidence regarding the use of CCDS systems.

The heterogeneity in the clinical criteria used, both for the CCDS system and the gold standard definitions, can be attributed to a lack of current consensus regarding pediatric identification, risk stratification, and diagnosis. Although the definition of adult sepsis was updated in 2016 [1], followed by the publication of the quick sepsis-related organ failure assessment tool [90], the most recent pediatric sepsis consensus definition was in 2005 [12] and has exhibited numerous limitations [31,91,92]. An extensive study by Weiss et al [93] found an interrater agreement of only 0.57 between the 2005 consensus and physician diagnosis of pediatric sepsis, further emphasizing the inadequacies of the current consensus criteria in practice. Researchers have since attempted to adapt the quick sepsis-related organ failure assessment to the pediatric population or pediatric logistic organ dysfunction-2, a pediatric deterioration tool, to sepsis [29,30,94,95]. Preliminary results from these studies show promise, demonstrating moderate to high prognostic accuracy for poor patient outcomes, such as mortality and pediatric intensive care unit admission [29,30,94,95]. Critical to this challenge is the unique pathophysiology of pediatric sepsis, in which simply age-adjusting adult sepsis criteria is controversial and inadequate [91,96]. For example, hypotension is commonly used as a key indicator of septic shock in adults; however, it is less useful in children, as hypotension is typically not present until much later in the disease course [25,26,91]. In addition, symptoms considered key to adult sepsis identification, such as tachycardia and tachypnea, are common in febrile children regardless of disease severity and can often be present due to crying and distress [25,26,95]. Therefore, there have been numerous calls by both academics and clinicians for an updated pediatric consensus in recent years [13,43,91,95]. In 2019, the Society of Critical Care Medicine convened the Pediatric Sepsis Definition Taskforce to update the consensus criteria for pediatric sepsis identification [97]. Although they have recently published a systematic review investigating the individual factors, clinical criteria, or illness severity scores that are used to identify children with sepsis who are at higher risk of developing organ dysfunction or death, the task force has not yet released an updated definition [97]. The absence of an up-to-date consensus for defining or detecting pediatric sepsis has likely contributed to the high diversity of CCDS clinical criteria used in pediatric populations and the range of definitions used for gold standard pediatric sepsis detection. Our findings demonstrate the need for more robust evidence to investigate the appropriate clinical criteria for pediatric sepsis and reinforce the urgent need for an updated consensus on the definition of pediatric sepsis.

Notably, an updated pediatric consensus must consider the extensive chronological and developmental age-dependent variability found in the pediatric population. For example, the pathophysiology of sepsis is expected to differ significantly among an adolescent, a child aged 5 years, and an infant aged 2 months. This will likely affect how different pediatric age groups present with sepsis, and accounting for these changes may not be as simple as adjusting the normal threshold of different vital signs according to age. This diversity needs to be studied and reflected in future consensus definitions and clinical criteria of the CCDS system.

### Neonatal Sepsis

Our findings report considerable variation across neonatal studies, despite most studies evaluating the same CCDS system: the Kaiser Permanente early-onset sepsis risk calculator (KPC) [89]. In particular, the gestational age of the neonates included in the study varied considerably (Table 4). Most studies investigated moderate to late preterm and term infants, with cutoffs for gestational age ranging from ≥34 to >37 weeks [98] or infants within their first month of life. A single study [69] investigated very preterm infants at <33 weeks gestational age [98], indicating a key research gap, as preterm infants are at a considerably higher risk of sepsis and infection than full-term newborns [14,28,32,99]. A recent study [99] demonstrated that more than one-third (38%) of extremely preterm infants, defined as infants ≤28 weeks’ gestation, had late-onset sepsis. The included studies investigated a diverse range of outcomes, related interventions, and responding personnel. Large multisite studies would improve the generalizability of the literature and thus should be considered despite the substantial difficulty in undertaking them.

Of the 18 neonatal studies included in this review, 14 (78%) investigated KPC [89]. This calculator combines the baseline early-onset sepsis incidence with maternal and infant characteristics and a clinical evaluation [89]. It aims to identify neonates at risk of early-onset sepsis, defined as sepsis within the first 72 hours after birth [28,87,88]. Under conventional sepsis management guidelines, many neonates are given potentially unnecessary antibiotic therapy as a precaution against sepsis, resulting in unintended negative effects [14,87]. A systematic review and meta-analysis performed by Achten et al [100] demonstrated that the use of KPC was associated with a reduction in antibiotic use. However, a more recent meta-analysis [101] showed that the KPC missed many cases of early-onset sepsis compared with the UK National Institute for Health and Care Excellence guidelines. This results in delayed or missed treatment for these neonates and suggests that further evaluation of the calculator is required [101]. In addition, the KPC is only designed for predicting sepsis risk in infants born at ≥34 weeks’ gestation within a very narrow early-onset sepsis time frame [87-89]. Our review identified only 17% (3/18) of neonatal studies that did not examine early-onset sepsis, with 6% (1/18) investigating late-onset sepsis and 11% (2/18) investigating general neonatal sepsis. Late-onset neonatal sepsis, often defined as sepsis occurring ≥3 days after birth, is a leading cause of mortality in vulnerable preterm infants [28,32,99,102]. This calls attention to a clear knowledge gap for future research into CCDS systems for neonatal sepsis occurring outside the initial 72 hours of life.

To date, no consensus definition has been developed for neonatal sepsis [15,16,28,103]. As the neonatal population is uniquely different from adults and older children, current adult and pediatric clinical criteria cannot be simply adapted [15,32,103]. A recently published systematic review [16] highlighted the variance in the currently used definitions of neonatal sepsis in randomized controlled trials. Surprisingly, the most commonly used definition was microbiological culture by itself or in combination with clinical signs and symptoms, despite the proven low sensitivity of this method and the high incidence of culture-negative sepsis among the neonatal population [14,16,102]. Similarly, some studies included in this review required a positive culture test to diagnose neonatal sepsis. A consensus on the definition of neonatal sepsis is needed to better identify suspected neonatal sepsis in clinical practice, for research studies, and to improve antibiotic stewardship in newborns [14,15,28,103]. Furthermore, any consensus criterion must acknowledge the age-related variability inherent to the neonatal population, as sepsis pathophysiology differs considerably between a preterm neonate and an infant in their first month of life [103].

### Maternal Sepsis

Despite the devastating consequences of sepsis in pregnant and immediately postpartum women [3,17,18], our comprehensive literature search identified only 2 studies that evaluated the use of CCDS systems for maternal sepsis. Pregnancy involves extensive physiological, hormonal, and psychological changes, which may mask the common symptoms of sepsis, resulting in delayed diagnosis and treatment [3,19,104]. A systematic review by Bauer et al [104] demonstrated that healthy pregnant women during the second and third trimesters often demonstrate considerable overlap with the SIRS criteria. This alteration of the usual physiological state must be represented in CCDS systems to ensure that sepsis in pregnant and immediately postpartum women is detected early, without the risk of unnecessary treatment in healthy patients. The lack of high-quality peer-reviewed studies in this population underlines a concerning knowledge gap in the literature, for which further research is urgently needed.

### Usability and Cost of CCDS Systems

The usability of any health intervention technology is critical for its successful implementation [105-108]. Therefore, investigating the usability of CCDS systems is essential for developing efficient and functional systems. In particular, alarm fatigue is a well-established usability concern for CCDS systems [109]. Alarm fatigue occurs when clinicians become desensitized to frequent inappropriate alarms and begin ignoring or overriding alerts, reducing the effectiveness of alert systems and potentially impacting patient outcomes [109,110]. To prevent alarm fatigue, CCDS systems must be carefully calibrated to avoid unnecessary frequent alerting [109,110]. None of the studies reported in this review investigated alarm fatigue in response to the implemented CCDS system, despite its importance for successful CCDS use.

Understanding the cost or cost-effectiveness of an intervention supports policy and clinical decision-making when determining resource allocation under limited health care budgets [111]. This is especially true for sepsis, which represents a large financial burden on the health system through both acute hospital care and long-term treatment and rehabilitation [112,113]. Of the 33 studies included in this review, only 4 (12%) investigated outcomes related to cost or usability, 1 (3%) in pediatric and 3 (9%) in neonatal populations, demonstrating a clear evidence gap for future research.

### Strengths and Limitations

This review comprehensively searched the available literature, both peer reviewed and gray, on the use of CCDS systems for inpatients with neonatal, pediatric, and maternal sepsis. Owing to time and resource constraints, the searches were limited to studies available in the English language and thus may have missed publications in other languages. Furthermore, the data extraction was performed by only 1 reviewer (KA). To limit any consequential data entry errors, the extraction form was extensively piloted, and any issues were cross-checked and fully discussed with the review team.

### Conclusions

Our findings have illustrated a comparative scarcity of studies investigating CCDS systems in pediatric, neonatal, and maternal inpatients, despite their high sepsis burden. Further research is needed to evaluate CCDS systems for the early detection of sepsis in these vulnerable populations. We identified extensive variation in the clinical criteria and gold standard definitions used by pediatric CCDS systems, and our findings reinforce calls for updated pediatric and neonatal sepsis consensus definitions. The review also shows a clear absence of studies investigating CCDS systems for sepsis identification in maternal inpatients, high-risk preterm populations, and neonates outside the first 72 hours of life. Finally, our review demonstrated a lack of studies investigating the usability and cost of CCDS systems, both of which are key to their effectiveness and sustainability. In conclusion, our review has identified substantial and important knowledge gaps in the literature evaluating CCDS systems for the early detection of sepsis in pediatric, neonatal, and maternal populations, which would benefit greatly from future research.

## Appendix

Multimedia Appendix 1 PRISMA-ScR (Preferred Reporting Items for Systematic Reviews and Meta-Analyses Extension for Scoping Reviews) checklist.

Multimedia Appendix 2 MEDLINE search strategy.

Multimedia Appendix 3 Adjustments made to data charting form.

Multimedia Appendix 4 Definitions of categories combining multiple subgroups.

Multimedia Appendix 5 Main study characteristics table.

Multimedia Appendix 6 Study setting by country.

## Abbreviations

CCDS: computerized clinician decision support
KPC: Kaiser Permanente early-onset sepsis risk calculator
LILACS: Latin American and Caribbean Health Sciences Literature
PQDT: ProQuest Dissertations and Theses Global
PRISMA: Preferred Reporting Items for Systematic Reviews and Meta-Analyses
PRISMA-ScR: Preferred Reporting Items for Systematic Reviews and Meta-Analyses Extension for Scoping Reviews
