# Supplementary material for: Computerized Clinical Decision Support Systems for the Early Detection of Sepsis Among Pediatric, Neonatal, and Maternal Inpatients: Scoping Review
Source: JMIR Med Inform. 2022 May 6;10(5):e35061. doi: 10.2196/35061 (PMC9123549; doi:10.2196/35061)
Supplement: Multimedia Appendix 3 [file medinform_v10i5e35061_app3.pdf]

Multimedia appendix 3 – Adjustments made to data charting form

1. Fields added to the form:
    a. Funding for the study, including the funding source (i.e. commercial/non-commercial)
    b. Conflicts of interest or financial disclosures
    c. Study length in months and start year
    d. The number of sites and notes on the sites
    e. The number of participants
    f. The type of sepsis investigated, e.g. early onset sepsis, septic shock etc.
    g. Any reporting guidelines followed. Since only one study described the use of reporting guidelines we also added 'clarity of outcomes reporting'. Studies were categorized as having 'good' clarity of outcome reporting if they specified the primary outcomes, the outcome analysis method, and the outcome measure definitions, and 'poor' clarity if the outcomes were not clearly described or there was a substantial reporting discrepancy between the methods and results. Studies were categorized as having 'average' clarity if they fulfilled some criteria of both good and poor.
    h. Reporting of any comorbidities of the included population
    i. EHR system
    j. If the alert was run silently, where the system was implemented but alerts were not delivered to clinicians, or was live, where alerts were delivered.
    k. We expanded vital sign criteria to include not only the general criteria, but also the specific clinical, infection-related, and organ dysfunction-related thresholds used by the system.
    l. Whether an outcome was a primary outcome, and the analysis method.
    m. Method of patient identification was charted only for studies examining sepsis identification as an outcome. In these studies, we additionally collected the gold standard definition used to identify sepsis.
2. Fields removed from form:
    a. Study power
    b. Location of CCDS implementation within the hospital electronic infrastructure.
3. A flowchart was used to categorize the study design for each study, presented in figure 1 below. The flowchart was designed and piloted prior to data charting.

Figure 1: Principal study categorization flow diagram

This flow chart was designed using reference material provided in appendix B of an Agency for Healthcare Research and Quality report[45] and articles by Ranganathan & Aggarwal[46-50].

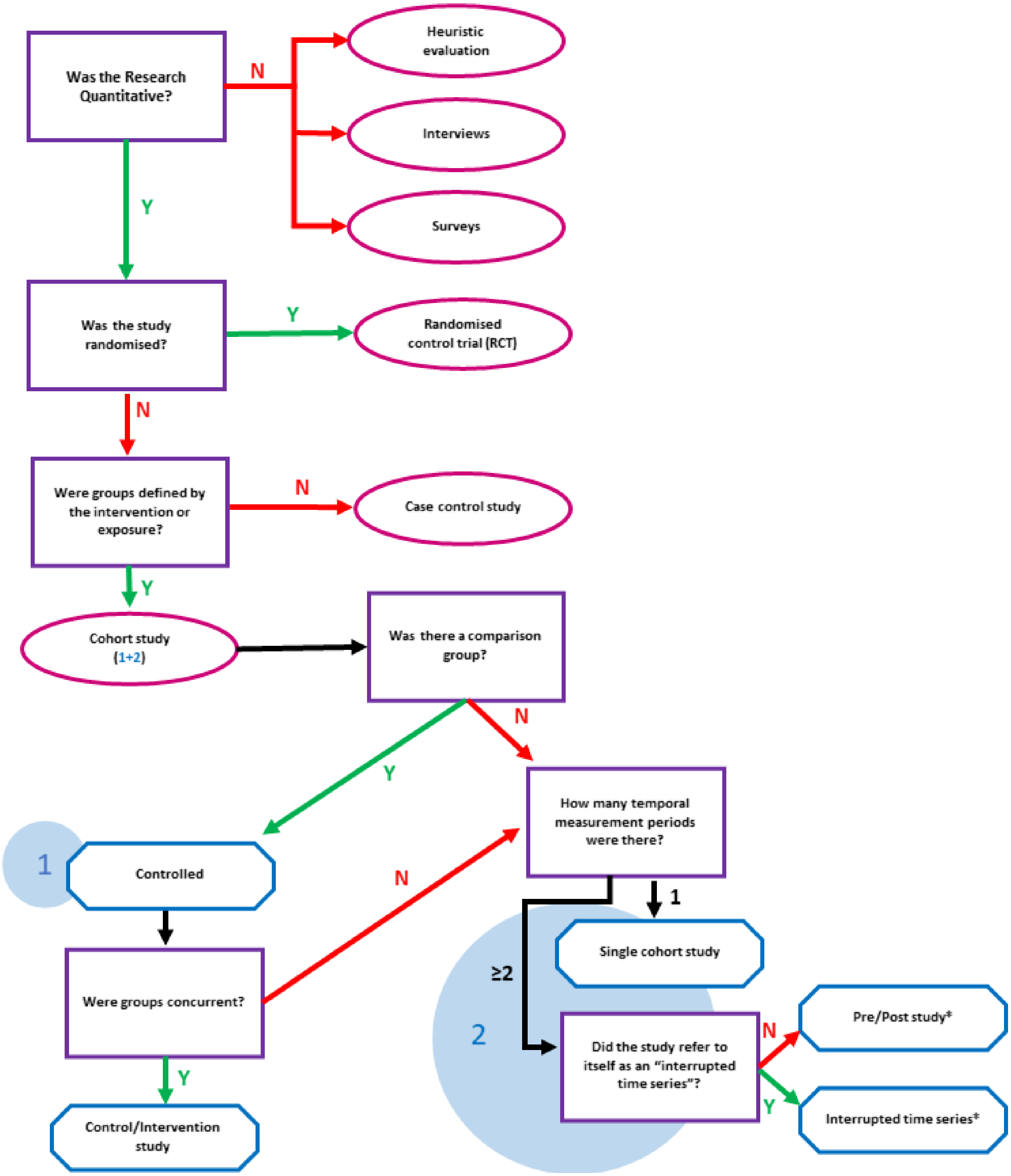

*Classification of study as before/after or interrupted time series will be slightly adjusted based on the individual paper methods.

4. The definition of the usability outcome category was modified to be defined as: the "extent to which a system, product or service can be used by specified users to achieve specified goals with effectiveness, efficiency, and satisfaction in a specific context of use" in keeping with the definition from the International Organisation for Standardization (ISO) in ISO 9241-11:2018 section 3.1.1[51]. Accordingly, we reported usability outcomes into either CCDS system effectiveness, efficiency, or satisfaction with the CCDS system. We required usability outcomes to be investigated from the end-user perspective (e.g., clinicians).

References

45. Viswanathan M, Berkman ND, Dryden DM, Hartling L. Assessing risk of bias and confounding in observational studies of interventions or exposures: further development of the RTI item bank. In: AHRQ Methods for Effective Health Care. Rockville (MD): Agency for Healthcare Research and Quality (US); 2013.
46. Ranganathan P, Aggarwal R. Study designs: Part 1 – An overview and classification. *Perspectives in Clinical Research.* 2018;9(4):184-186. PMID:30319950
47. Aggarwal R, Ranganathan P. Study designs: Part 2 – Descriptive studies. *Perspectives in Clinical Research.* 2019;10(1):34-36. PMID:30834206
48. Ranganathan P, Aggarwal R. Study designs: Part 3 - Analytical observational studies. *Perspectives in Clinical Research.* 2019;10(2):91-94. PMID:31008076
49. Aggarwal R, Ranganathan P. Study designs: Part 4 – Interventional studies. *Perspectives in Clinical Research.* 2019;10(3):137-139. PMID:31404185
50. Aggarwal R, Ranganathan P. Study designs: Part 5 – interventional studies (II). *Perspectives in Clinical Research.* 2019;10(4):183-186. PMID:31649869
51. Ergonomics of human-system interaction — Part 11: Usability: Definitions and concepts. ISO Standard No. 9241-11:2018(en). The International Organization for Standardization. 2018. URL: https://www.iso.org/obp/ui/#iso:std:iso:9241:-11:ed-2:v1:en [accessed 2021-02-26]
