# Supplementary material for: Computerized Clinical Decision Support Systems for the Early Detection of Sepsis Among Pediatric, Neonatal, and Maternal Inpatients: Scoping Review
Source: JMIR Med Inform. 2022 May 6;10(5):e35061. doi: 10.2196/35061 (PMC9123549; doi:10.2196/35061)
Supplement: Multimedia Appendix 4 [file medinform_v10i5e35061_app4.pdf]

Multimedia appendix 4: Definitions of categories combining multiple subgroups

Table 1 – Context and outcome characteristics for pediatric studies

Inpatient wards = combines acute care unit, inpatients, and inpatient units (not intensive care units (ICU) or cardiology units) groups

ED = combines the emergency department (ED) and pediatric ED groups

<u>Patient outcomes</u>

Sepsis identification = combines severe sepsis identification (treatment for severe sepsis), shock identification, and sepsis identification groups.

Clinician discretion = combines treated as sepsis by care team: 1) documentation of concern for sepsis, 2) use of sepsis order set, 3) at least 48hours of intravenous (IV) antibiotics, clinical assessment of ED attending physician, documentation of sepsis/suspected sepsis in chart, clinician/trained nurse verified, and initiation of sepsis treatment groups.

Other = combines mortality (total inpatient), pediatric ICU (PICU) admission, length of stay (hospital), length of stay (PICU), total number of admissions, need for respiratory support, patient location, and need for vasoactive support groups.

<u>Sepsis treatment or management</u>

Timeliness of alert or interventions = combines time to antibiotics, time to first positive screen or alert, timeliness of alert, time to blood culture, average time to antibiotics, and median time to antibiotics groups.

Other = combines proportion of untreated sepsis patients, proportion of patients with severe sepsis screened in ED, number of diagnostic/therapeutic interventions, days between delayed sepsis recognition, rapid fluid bolus given within 20mins, lactate measurement within 1hr, shock score and pediatric early warning score (PEWS) at time of shock huddle alert, antibiotics given within 1hr, and sepsis management recommendations followed (lactate checked & antibiotics given within 60mins, and fluid bolus given within 20 minutes groups.

Figure 2 – Studies by year, population, and publication type

Conference abstract = combines the conference abstract and Q-tip groups.

Journal article = combines the journal article and short communication groups.

Figure 3 – Proportion of studies reporting each outcome category

Conference abstract = combines the conference abstract and Q-tip groups.

Journal article = combines the journal article and short communication groups.

Pediatric in text

Chart review = combines chart review and manual review groups.

Prospectively screened patients = combines prospective study and daily screen of all PICU patients' groups.

Table 2 - Computerized clinical decision support (CCDS) characteristics in pediatric studies

Sepsis = combines pediatric sepsis, sepsis, sepsis/suspected sepsis, and sepsis and septic shock groups.

Severe sepsis = combines severe sepsis and severe sepsis/septic shock groups.

Both = Both silent and live in different stages of the before/after study.

Response team (related interventions) = combines sepsis huddle and response team groups.

Education & information resources = combines education, help cards, question & answer (Q&A), training simulations, and feedback to providers groups.

Other = combines event note, new specific clinician position, downstream provider alert, and sepsis notice board groups.

Nurses = combines nurses and nurse practitioner groups.

Other clinicians = combines providers, physician, and clinician groups.

Response team (responding personnel) = combines response team, and paediatric critical care team groups.

EHR = combines electronic health record (EHR) and EHR prompt box groups.

Table 4 – Context and outcome characteristics for neonatal studies

Conference abstract = combines the conference abstract and Q-tip groups

Journal article = combines the journal article and short communication groups

Hospital-wide = combines the hospital-wide and 'cardiac critical care unit, inpatients and neonatal ICU (NICU)' groups

Nursery = combines the 'NICU and nursery', newborn nursery, neonatal unit, mother/baby units, and perinatal center groups.

Patient outcomes

ICU admission = combines NICU admission, NICU admission (maternal chorioamnionitis (MC) exposed infants), and neonatal unit admissions groups.

Length of stay (LOS) = combines LOS, LOS (hospital), and hours in NICU groups.

Other = combines estimated early onset sepsis risk at start of antibiotic treatment, readmission rate within 28 days after discharge, readmission rates, missed cases of early onset sepsis, admissions, and late onset sepsis detection groups.

Sepsis treatment or management

Timeliness of alert or interventions = combines time to treatment start and late onset sepsis detection timing groups.

Other = combines interventions begun within 2hrs of alert, overlap and discrepancies between current Centers for Disease Control and Prevention (CDC)/American Academy of Pediatrics (AAP) guidelines and Kaiser Permanente Sepsis Risk Calculator recommendations,

documentation of calculator scores, type of intervention, impact of incorrect calculation (change in clinical decision), and relevant clinical interventions performed within 2hrs groups

Antibiotics = combines antibiotic duration, antibiotic prescription rate (within 72 hours of life), antibiotics reception by chorioamnionitis-exposed neonates, antibiotics given to newborns with a blood culture drawn, use of antibiotics for suspected early onset sepsis, extension of antibiotics beyond 72 hours of life, antibiotics given ≤72hours, 36 hours of antibiotics given, antibiotic days/1000 patient admission days, antibiotics given, antibiotics doses received in first 48 hours, antibiotic utilization rate in first 48 hours, infant antibiotic orders, 5 days of antibiotics given, antibiotic days, number of gentamicin serum concentration tests, and monthly antibiotic initiation rates groups.

Laboratory evaluation = combines late positive blood culture (obtained between 72 hours of life and 7 days), blood culture obtained for early onset sepsis evaluation (within 72 hours of life), "late" blood cultures (obtained between 72 hours of life and 7 days), number of blood cultures drawn for neonates delivered to mothers with chorioamnionitis, laboratory tests, number of complete blood counts (CBC) ordered, lab evaluation rate in first 48 hours, blood culture order rate, reason for blood culture, monthly sepsis evaluation rates, C-reactive protein (CRP) order rate, CBC order rate, blood cultures ordered, Blood culture and CBC and CRP tests, blood culture and CBC tests, blood culture only, CBC and CRP tests, CBC only, repeat sepsis evaluations (between 72 hours of life and 7 days), number of blood cultures, number of CRP tests, number of blood counts, and laboratory evaluation of chorioamnionitis-exposed neonates groups.

Sepsis guideline compliance = combines adherence to sepsis calculator recommendation, and early onset sepsis calculator compliance groups.

Cost = combines total charges, and total bed charges groups.

Table 5 - Computerized clinical decision support (CCDS) characteristics in neonatal studies

Conference abstract = combines the conference abstract and Q-tip groups

Journal article = combines the journal article and short communication groups

Both = Both silent and live in different stages of the before/after study

Education & information resources = combines education, email alert & resources about tool, Q&A, feedback to providers, help cards, and feedback on program groups

Other (related interventions) = combines reminders, antibiotic time-out, updated guidelines, key-driver diagram & outcome goals, scores for central line-associated blood stream infections (CLABSI), catheter-associated urinary tract infections (CAUTI), and pediatric early warning systems (PEWS), note templates, and quality improvement program groups

Nurses = combines nurse practitioner, and nurse groups

Other clinicians = combines physician, pediatric and family medicine residents, pediatrician, providers, neonatal team member, medical personnel, senior level fellows, and resident groups.

Other (alert delivery) = combines color-coded score on monitor in logistics center and automatically populated resident sign out sheet groups.
