# Supplementary material for: Computerized Clinical Decision Support Systems for the Early Detection of Sepsis Among Pediatric, Neonatal, and Maternal Inpatients: Scoping Review
Source: JMIR Med Inform. 2022 May 6;10(5):e35061. doi: 10.2196/35061 (PMC9123549; doi:10.2196/35061)
Supplement: Multimedia Appendix 5 [file medinform_v10i5e35061_app5.pdf]

Multimedia Appendix 5 - Main characteristics Table

Pediatric

Author (Year)	Type of publication	Country	Principal study type	Setting	# of sites	# of participants	Type of sepsis	CCDS type	Clinical criteria (general)	Outcome category
Balamuth (2017) [55]	Journal article	United States	Pre/post (not controlled)	Paediatric ED	1	182,509	Severe sepsis/septic shock	Homegrown	Abnormal heart rate or hypotension AND infection AND one or more of abnormal capillary refill, abnormal mental status, or high-risk condition	Patient outcomes, sepsis treatment/management
Coffman (2018) [58]	Conference abstract	United States	Pre/post (not controlled)	Acute care unit	1	NR	Sepsis	Homegrown	Abnormal temperature AND (PEWS change or family concern or vital sign changes or patient risk factors)	Sepsis treatment/management, usability
Cruz (2012) [59]	Journal article	United States	Single cohort	ED	1	39,697	Septic shock	Homegrown	(Temperature abnormality or abnormal pulse after corrected for pyrexia or abnormal heartrate) AND (high risk patient or ill-appearing child (poor perfusion or mentation))	Patient outcomes
Dewan (2020) [61]	Journal article	United States	Single cohort	PICU	1	424	Sepsis	Homegrown	Abnormal temperature AND blood culture order AND (delayed capillary refill or cool pale skin or altered mental state or weak pulses or flash capillary refill or bounding pulses or hypotension)	Patient outcomes, sepsis treatment/management
Eisenberg (2021) [64]	Journal article	Unspecified	Pre/post (not controlled)	Paediatric ED	1	122,221	Severe sepsis/septic shock	Unspecified	CLINICIAN-INITIATED ALERT: (fever or suspected infection) AND 3 or more of abnormal temperature, blood pressure, heart rate, respiratory rate, capillary refill, mental status, pulse quality, skin, or high-risk condition. AUTOMATIC ALERT: 3 stages - SIRS alert: 2 or more of abnormal white blood cell count, temperature, heart rate, or respiratory rate (1 must be white blood cell count or temperature), Sepsis alert: SIRS alert AND 1 noncardiac organ dysfunction, Severe sepsis alert: SIRS alert AND (cardiac or 2 other organ dysfunctions).	Patient outcomes, sepsis treatment/management
Lloyd (2018) [71]	Journal article	United States	Single cohort	Paediatric ED	1	29	Sepsis	Homegrown	(4 of abnormal heart rate or respiratory rate or systolic blood pressure or capillary refill or temperature or mental status or pulse or skin (3 if the patient has a high-risk condition)) AND concern for infection/historical temperature abnormality	Patient outcomes, sepsis treatment/management

Mangubat (2014) [73]	Conference abstract	Unspecified	Pre/post (not controlled)	Hospital-wide	1	449	Severe sepsis	Homegrown	NR	Patient outcomes
Salomon (2016) [74]	Conference abstract	United States	Single cohort	ED	1	47,272	Paediatric sepsis	Homegrown	Algorithm of 9 clinical parameters	Patient outcomes
Stinson (2019) [77]	Journal article	United States	Pre/post (not controlled)	Inpatient units (not ICU or cardiology units)	1	20,420	Severe sepsis/septic shock	Homegrown	Score out of 110, alerts at ≥ 45 points. Points for: a high-risk condition or abnormal mental status, capillary refill, pulse, temperature, skin, or tachycardia or tachypnoea or hypotension.	Patient outcomes, sepsis treatment/management
Torres (2015) [80]	Conference abstract	United States	Single cohort	Inpatients	1	70	Sepsis/suspected sepsis	Commercial	American Academy of Pediatrics septic shock collaborative shock tool score ≥ 25	Patient outcomes
Vidrine (2020) [81]	Journal article	United States	Pre/post (not controlled)	PICU	1	NR	Sepsis	Homegrown	Abnormal temperature AND blood culture order AND (delayed capillary refill or cool pale skin or altered mental state or weak pulses or flash capillary refill or bounding pulses or hypotension)	Sepsis treatment/management
Viteri (2018) [82]	Conference abstract	United States	Single cohort	Inpatients	1	42	Sepsis and septic shock	Homegrown	Abnormal vital signs and clinical findings (score ranges from 0-110, alerts ≥ 45)	Sepsis treatment/management
West (2018) [83]	Conference abstract	United States	Single cohort	Hospital-wide	1	3,299	Severe sepsis	Homegrown	NR	Patient outcomes

ED = Emergency department, NR = Not reported, PEWS = Pediatric emergency warning score, PICU = Pediatric intensive care unit, SIRS = systemic inflammatory response syndrome, ICU = intensive care unit.

Neonatal

Author (Year)	Type of Publication	Country	Principal Study type	Setting	# of sites	# of participants	Specific age range	Type of sepsis	Clinical criteria (general)	Outcome category
Achten (2018) [52]	Journal article	Netherlands	Pre/post (not controlled)	Hospital-wide	1	3,953	≥35 weeks gestation	Early onset sepsis	Kaiser Permanente early onset sepsis risk calculator	Patient outcomes, sepsis treatment/management
Achten (2020) [53]	Journal article	Netherlands	Pre/post (not controlled)	Mother/ baby units, neonatal unit	1	1,708	≥35 weeks gestation	Early onset sepsis	Kaiser Permanente early onset sepsis risk calculator	Patient outcomes, sepsis treatment/management, cost
Arora (2019) [54]	Journal article	Unspecified	Pre/post (not controlled)	NICU	1	539	≥ 34 weeks gestation	Early onset sepsis	Kaiser Permanente early onset sepsis risk calculator	Sepsis treatment/management
Beavers (2018) [56]	Journal article	United States	Single cohort	NICU	1	258	Unspecified	Early onset sepsis	Kaiser Permanente early onset sepsis risk calculator	Patient outcomes, sepsis treatment/management, cost
Dhudasia (2018) [62]	Journal article	United States	Interrupted time series (not controlled)	Hospital-wide	1	11,782	≥36 weeks gestation	Early onset sepsis	Kaiser Permanente early onset sepsis risk calculator	Sepsis treatment/management

Eason (2021) [63]	Journal article	United Kingdom	Pre/post (not controlled)	Neonatal unit	1	1,725	>37 weeks ("term infants")	Early onset sepsis	Kaiser Permanente early onset sepsis risk calculator	Sepsis treatment/management
Emmanuel (2018) [65]	Journal article	United States	Single cohort	cardiac critical care unit, inpatients, NICU, PICU	1	NR	First month of life	Neonatal sepsis	Age AND abnormal temperature AND antibiotic use	Sepsis treatment/management
Fowler (2019) [66]	Journal article	United States	Pre/post (not controlled)	NICU, nursery	4	252	≥ 34 weeks gestation	Early onset sepsis	Kaiser Permanente early onset sepsis risk calculator	Sepsis treatment/management, usability
Gievers (2018) [67]	Journal article	United States	Pre/post (not controlled)	Hospital-wide	1	356	≥35 weeks gestation	Early onset sepsis	Kaiser Permanente early onset sepsis risk calculator	Patient outcomes, sepsis treatment/management
Goyack (2015) [68]	Conference abstract	United States	Single cohort	Hospital-wide	1	NR	Unspecified	Neonatal sepsis	Indicators of infection (CLABSI, CAUTI, neonatal sepsis) or worsening clinical condition (PEWS)	Sepsis treatment/management
Gur (2015) [69]	Journal article	Israel	Single cohort	NICU	2	118	< 33 weeks	Late onset sepsis	RALIS: abnormal heart rate, respiratory rate, core body temperature, body weight, documented low oxygen saturation, and documented bradycardia (score out of 10)	Patient outcomes, sepsis treatment/management
Klingaman (2018) [70]	Q-tip	United States	Single cohort	Newborn nursery	1	505	Unspecified	Early onset sepsis	Kaiser Permanente early onset sepsis risk calculator	Sepsis treatment/management
Mahdally (2018) [72]	Conference abstract	Unspecified	Single cohort	Hospital-wide	1	NR	≥ 34 weeks gestation	Early onset sepsis	Maternal risk factors AND hospital-specific early onset sepsis incidence AND infant vital signs	Sepsis treatment/management
Sharma (2019) [75]	Journal article	United States	Pre/post (not controlled)	NICU, nursery	1	5,346	≥36 weeks gestation	Early onset sepsis	Kaiser Permanente early onset sepsis risk calculator	Patient outcomes, sepsis treatment/management
Skey (2018) [76]	Conference abstract	United States	Pre/post (not controlled)	Newborn nursery	1	51	≥35 weeks gestation	Early onset sepsis	Kaiser Permanente early onset sepsis risk calculator	Patient outcomes, sepsis treatment/management
Stipelman (2019) [78]	Journal article	United States	Interrupted time series (not controlled)	Newborn nursery	1	11,924	Unspecified	Early onset sepsis	Kaiser Permanente early onset sepsis risk calculator	Sepsis treatment/management
Strunk (2018) [79]	Short communication	Australia	Pre/post (not controlled)	Perinatal centre	1	4,234	≥35 weeks gestation	Early onset sepsis	Kaiser Permanente early onset sepsis risk calculator	Patient outcomes, sepsis treatment/management
Zayek (2020) [84]	Journal article	United States	Pre/post (not controlled)	Newborn nursery	1	4,053	≥ 34 weeks gestation	Early onset sepsis	Kaiser Permanente early onset sepsis risk calculator	Sepsis treatment/management

NICU = Neonatal intensive care unit, PICU = Pediatric intensive care unit, NR = Not reported, CLABSI = Central line-associated blood stream infections, CAUTI = Catheter-associated urinary tract infections, PEWS = pediatric early warning system.

Maternal

Author (Year)	Type of Publication	Country	Principal Study type	Setting	# of sites	# of participants	Type of sepsis	CCDS Type	Clinical criteria (general)	Outcome category
Blumenthal (2020) [57]	Conference abstract	Unspecified	Pre/post (not controlled)	Hospital-wide	3	NR	Obstetric	Unspecified	Abnormal temperature AND (heart rate or altered mental status or respiratory rate or mean arterial pressure sustained over 10 minutes)	Patient outcomes, sepsis treatment/management
Davis (2018) [60]	Abstract	United States	Insufficient information to determine	Hospital-wide	1	NR	Obstetric	Homegrown	2 Obstetric adjusted SIRS AND 1 Organ dysfunction	Sepsis treatment/management

NR = Not reported, SIRS = systemic inflammatory response syndrome.
