# Supplementary material for: Computerized Clinical Decision Support Systems for the Early Detection of Sepsis Among Pediatric, Neonatal, and Maternal Inpatients: Scoping Review
Source: JMIR Med Inform. 2022 May 6;10(5):e35061. doi: 10.2196/35061 (PMC9123549; doi:10.2196/35061)

Multimedia Appendix 6. A) Countries in which the pediatric studies are set. B) Countries in which the neonatal studies are set.

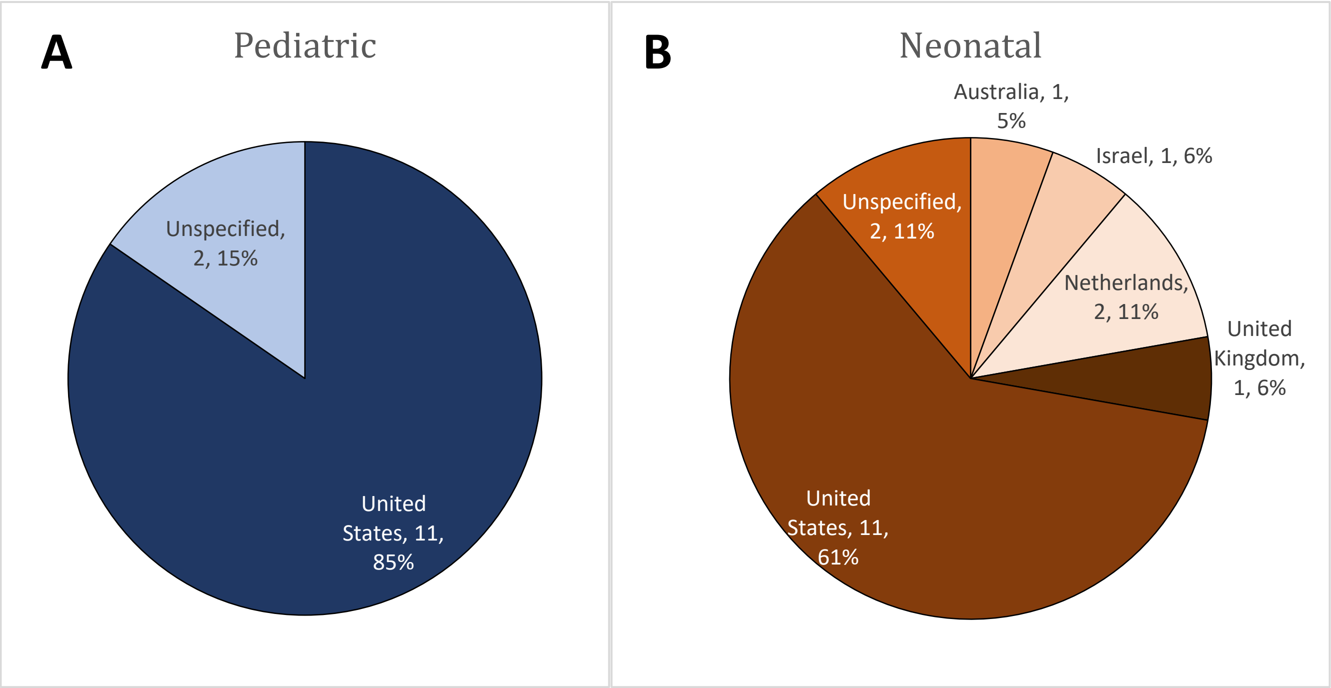

Supplement: Multimedia Appendix 6 [file medinform_v10i5e35061_app6.pdf]
